# Salud sexual y reproductiva de mujeres jóvenes migrantes de Latinoamérica y el Caribe que viven en Chile

**DOI:** 10.15446/rsap.V25n3.101507

**Published:** 2023-05-01

**Authors:** Ingrid Leal-Fuentes, Temístocles Molina-González, Carolina Carstens-Riveros

**Affiliations:** 1 IL: Matrona. M. Sc. Salud Pública. Universidad de Chile. Santiago de Chile, Chile. igleal@uchile.cl Universidad de Chile Salud Pública Universidad de Chile Santiago de Chile Chile igleal@uchile.cl; 2 TM: Estadístico. M. Sc. Bioestadística. Universidad de Chile. Santiago de Chile, Chile. tmolina@uchile.cl Universidad de Chile Bioestadística Universidad de Chile Santiago de Chile Chile tmolina@uchile.cl; 3 CC: Sociól. M. Sc. Antropología aplicada a Salud y Desarrollo Comunitario. Universidad de Chile. Santiago de Chile, Chile. ccarstens@uchile.cl Universidad de Chile Antropología aplicada a Salud y Desarrollo Comunitario Universidad de Chile Santiago de Chile Chile ccarstens@uchile.cl

**Keywords:** Salud sexual y reproductiva, análisis de género en salud, emigración, inmigración *(fuente: DeCS, BIREME)*, Sexual and reproductive health, gender analysis in health, emigration, immigration *(source: MeSH, NLM)*

## Abstract

**Objetivo:**

Analizar variables de salud sexual y reproductiva de mujeres adolescentes y jóvenes migrantes y chilenas.

**Métodos:**

Estudio transversal, analítico de la Novena Encuesta Nacional de la Juventud. La muestra correspondió a 3 140 mujeres de 24 años y menos, extranjeras y chilenas. Se realizó un análisis descriptivo y de asociación mediante prueba Rao-Scott. La asociación se midió calculando Odds ratio. Se ajustaron modelos de regresión lineal múltiple y logística múltiple.

**Resultados:**

Las jóvenes migrantes correspondieron al 3,47%. En promedio llegan a Chile a una edad de 17,58 años. Estas jóvenes presentaron mayor porcentaje de convivencia (21,49% vs. 10,67%, p=0,002), hijos (45,28% vs. 23,69%, p=0,002), mayor edad para embarazo no planificado (18,92 años vs. 17,67 años, p=0,033) y primera pareja sexual esposo o conviviente (19,47% vs. 6,09%, p=0,017). La razón entre las jóvenes que no practican sexo anal versus las que sí lo practican, es 2,6 (1/0,38) veces mayor en extranjeras (OR: 0,38; IC 95%: 0,16-0,91). La razón entre jóvenes que usaron un anticonceptivo menos efectivo en la primera y en la última relación sexual versus aquellas que usaron uno efectivo o más efectivo, es 2,19 y 2,40 veces mayor en las jóvenes migrantes que en las chilenas (OR: 2,19; IC 95%:1,03-4,64 vs. OR: 2,40; IC 95%:1,16-4,97).

**Conclusiones:**

Las jóvenes extranjeras son un grupo pequeño de la población joven que vive en Chile. Existen similitudes entre jóvenes migrantes y chilenas para acceder a anticonceptivos y diferencias en el tipo de pareja y el estado civil al iniciar su actividad sexual, el número de hijos, las prácticas sexuales y el tipo de anticonceptivo usado.

## Abstract (en)

**Objective:**

To analyze SRH variables of migrant and Chilean adolescent and young women.

**Methods:**

cross-sectional, analytical study of the 9th National Youth Survey. The sample consisted of 3,140 foreign and Chilean women aged 24 years old and younger. Descriptive and association analysis was performed using the Rao-Scott test. The association was measured by calculating odds ratio. Multiple linear, multiple logistic and multiple regression models were adjusted.

**Results:**

young migrant women are 3.47 °% of the sample. On average, they arrived in Chile at 17.58 years old. Young women in cohabitation and with children were mostly migrants (21.49% v/s 10.67%) (p=0.002), (45.28% v/s 23.69%) (p=0.002) and were older at the time of unplanned pregnancy (18.92 years v/s 17.67) (p=0.033). The first sexual partner, husband or cohabitant, was more frequent in foreign women (19.47% and 6.09%) (p=0.017). Foreign young women were 2.6 (OR: 0.38; 95% CI: 0.16 -0.91) times less likely to have anal sex and 2 times more likely to use a less effective contraceptive at first and last intercourse (OR: 2.19; 95 % CI: 1.03 - 4.64) (OR: 2.40; 95 % CI: 1.16 - 4.97), compared to Chilean women.

**Conclusions:**

Foreign young women are a small group within the young population living in Chile. There are similarities between migrants and Chilean women in terms of access to contraceptives, and differences in relation to the type of partner and marital status at the time of initiation of sexual relations, number of children, sexual practices and type of contraceptive used.

La migración es un fenómeno complejo y dinámico que se ha acentuado en los últimos años a nivel latinoamericano y mundial, siendo reconocida como un determinante social de salud 1,2. Las causas y las consecuencias de los desplazamientos migratorios son múltiples, se relacionan con aspectos económicos, laborales y legales, entre otros 2. Si bien las migraciones pueden abrir nuevas oportunidades, se viven de diferentes formas dependiendo de las características personales, el nivel socioeconómico y educacional, el género, el origen étnico, el estatus migratorio, el idioma, el país de origen y el de destino 3. Estas constituyen un reto para lograr la integración cultural entre las comunidades migrantes y las sociedades de acogida.

En Chile ha incrementado en los últimos años el fenómeno migratorio desde países latinoamericanos y del Caribe 4. La población extranjera que vive en el país es de aproximadamente 1,5 millones de personas; de estas, más del 70% provienen de Venezuela (30,5%), Perú (15,8%), Haití (12,5 %), Colombia (10,8 %) y Bolivia (8,0 %) 4.

Los adolescentes y jóvenes extranjeros menores de 25 años que viven en Chile llegan a 258 269, aproximadamente, y representan el 17% del total de la población extranjera en Chile. De estos, cerca del 50% son mujeres, lo que pone en evidencia la feminización del fenómeno migratorio 5. Si bien, los países de origen comparten características macroestructurales, también se presentan diferencias que se traducen en la diversidad del grupo 2,6.

Existe escasa investigación y políticas públicas que visibilicen las necesidades particulares de los adolescentes y jóvenes migrantes 7. En parte, porque los países de destino establecen estrategias de adecuación focalizadas en otros grupos, como la población infantil, considerada prioritaria dentro de la política social 8. Otras razones podrían estar relacionadas con la rápida incorporación al mundo adulto, ya sea a través del ámbito laboral o por la conformación de familia. Así, el concepto tradicional de adolescencia y juventud no necesariamente se aplica en este grupo 8,9.

La sexualidad de la gente joven históricamente ha sido tratada desde el adultocentrismo y el riesgo, especialmente hacia las mujeres. Las adolescentes y jóvenes extranjeras comparten características socioculturales con las chilenas, que impactan en su salud sexual y reproductiva (SSR) al estar insertas en la cultura latina tradicional, patriarcal y sexualmente conservadora 10. También comparten, de manera transversal, las barreras de acceso a los servicios de SSR. Otras dificultades son particulares de las jóvenes migrantes, como las idiomáticas, la falta de documentación y el desconocimiento del sistema de salud, a pesar de las acciones implementadas por el Gobierno para mejorar el acceso 7,9,11. Las adolescentes y jóvenes migrantes que viven en Chile también podrían enfrentarse a otras dificultades descritas en la literatura, asociadas a la aculturación e incorporación a una sociedad que puede ser percibida como individualista, etnocéntrica, capitalista y que no reconoce otras formas de vida 12. Esto sumado a los prejuicios en torno a su sexualidad, estereotipos de género y dificultades para ejercer sus derechos sexuales y reproductivos (DSR), las exponen a violencia sexual, embarazos no deseados e ITS/VIH 5,9,13,14,15. En contraste, otros estudios evidencian que las personas de primera generación tienen menos conductas de riesgo que las generaciones siguientes nacidas en el país de destino, probablemente porque la cultura de origen ejerce un efecto protector para conductas de riesgo 8,9,10,12,16,17.

En Chile la tasa específica de fecundidad adolescente (TFA) se encuentra en 26 nacimientos por cada 1 000 mujeres entre 15 y 19 años, muy por debajo del promedio de Latinoamérica y el Caribe de 66,5, 15,18,19. Por otro lado, la tasa global de fecundidad (TGF) en el país es de 1,6, mientras que en países de Latinoamérica y el Caribe, como Perú, Bolivia y Haití rodea el 2,5 20. Además, Haití y Bolivia tienen las prevalencias más bajas de uso de anticonceptivos (31,3% y 34,6%, respectivamente), mientras que Brasil, Colombia y Cuba tienen las prevalencias más altas (70%) 21. Estos datos tienen relación con las inequidades entre los países para el acceso a métodos anticonceptivos modernos, así como a la educación sexual integral, sin considerar las inequidades dentro de cada país.

Chile es el país latinoamericano que presenta un mayor incremento de casos nuevos de VIH, además de una feminización de la epidemia 22, tanto en población general como en adolescentes y jóvenes. Durante el 2019, el 40,3% de los casos nuevos confirmados correspondieron a personas extranjeras, por lo que han sido consideradas una población clave para estrategias de prevención y monitoreo 13,23.

Algunos informes muestran que la mayoría de las estrategias de salud para las mujeres migrantes se orientan al cuidado materno infantil, quienes presentarían peores resultados maternos y neonatales respecto de las mujeres chilenas 5,24. Sin embargo, estas estrategias no consideran aspectos como la regulación de la fertilidad y la sexualidad desde una perspectiva integral 5. Existe escasa evidencia a nivel nacional que describa las particularidades y diferencias en SSR de este grupo, por lo que se hace necesario explorar sus necesidades desde el enfoque de derechos 7. El objetivo es analizar variables de SSR de mujeres adolescentes y jóvenes migrantes y chilenas.

## MÉTODOS

Estudio transversal y analítico. La muestra se obtuvo de la base de datos de la Novena Encuesta Nacional de la Juventud. El período de recolección de datos fue de diciembre de 2018 a abril de 2019. El cálculo del tamaño muestral con significación nacional, el tipo de muestreo, su diseño, la selección de los participantes y cómo se recolectó la información se describe en el informe de la Novena Encuesta Nacional de Juventud 25. El factor de ponderación fue considerado en los análisis. Esta encuesta cuenta con 14 módulos temáticos. Para este estudio se seleccionaron cinco módulos temáticos: educación, pareja y familia, salud sexual y reproductiva, violencia y otros.

La muestra fue de 3 140 mujeres de 24 años y menos, extranjeras y chilenas, con y sin inicio de actividad sexual, provenientes de sectores urbanos y rurales. De esta muestra, para el análisis de variables reproductivas y algunas de sexualidad, se incluyeron sólo a aquellas sexualmente activas, correspondientes a 1 924 participantes.

La variable nacionalidad fue clasificada como extranjera, para aquellas adolescentes y jóvenes con nacionalidades de países de Latinoamérica y el Caribe, y chilena en el caso de adolescentes nacidas en Chile. Se excluyeron otras nacionalidades.

Las variables sociodemográficas fueron: edad en años cumplidos, nivel socioeconómico, nivel de estudios, situación de pareja y sistema previsional de salud.

Las variables reproductivas fueron: número de hijos, edad del primer embarazo, embarazo no planificado, edad del embarazo no planificado, uso de método anticonceptivo (MAC), primera relación sexual, uso de MAC en la última relación sexual, tipo de MAC usado en la primera relación sexual y tipo de MAC usado en la última relación sexual, según su efectividad 26.

Las variables de sexualidad fueron: inicio de actividad sexual, edad de inicio de la actividad sexual, número de parejas sexuales, uso de condón en la última relación sexual, antecedentes de violencia de pareja, antecedente de aborto provocado, sexo oral, sexo anal, tipo de relación con primera pareja sexual y tipo de relación con última pareja sexual.

### Análisis estadístico

Se trabajó con análisis estadístico para muestras ponderadas (survey). Asimismo, se realizó un análisis descriptivo de la información para caracterizar la muestra y se midió la asociación entre las variables sociodemográficas, reproductivas y de salud sexual, comparando población chilena con extranjera, mediante la prueba estadística de Rao-Scott. Se evaluó la asociación entre las variables edad de embarazo no planificado y nacionalidad, mediante el cálculo de coeficiente (Coef.) (modelo de regresión lineal múltiple); sexo anal y tipo de MAC usado en la primera y en la última relación sexual, y nacionalidad, mediante el cálculo de OR (odds ratio) ajustado por edad, nivel socioeconómico y nivel de educación, por medio del ajuste de tres modelos de regresión logística múltiple. Los datos del estudio fueron analizados utilizando el software estadístico Stata v 12 (StataCorp LP Texas, USA). Este estudio fue sometido al Comité de Ética de Investigación en Seres Humanos de la Facultad de Medicina de la Universidad de Chile.

## RESULTADOS

Las jóvenes migrantes correspondieron al 3,47% de la muestra, las nacionalidades fueron en su mayoría peruana (25,24%) y venezolana (22,68%). En promedio llegaron a los 17,58 años de edad a Chile y llevan residiendo 2,24 años. El 56,91% tenía 19 años o menos al llegar a Chile y el 64,42% llevaba viviendo en el país dos años o menos (Tabla 1).

**Tabla 1:** Distribución de nacionalidades, edad de ingreso y tiempo de residencia de jóvenes y adolescentes

Nacionalidad	Prevalencias %
Chilena	96,53
Extranjera	3,47
Total	100
Extranjera	
Argentina	2,54
Boliviana	10,64
Brasilera	0,5
Colombiana	13,59
Cubana	0,62
Dominicana	15,26
Haitiana	8,48
Peruana	25,24
Venezolana	22,68
Paraguaya	0,45
Total	100
Edad promedio de llegada a Chile de las jóvenes migrantes	17,58
(IC 95 %)	(15,67-19,48)
Tiempo promedio que llevan en Chile las jóvenes migrantes	2,24
(IC 95 %)	(1,45-3,03)

Con respecto a las variables sociodemográficas, no se encontraron diferencias estadísticamente significativas en cuanto a edad y nivel socioeconómico. En ambos grupos, la mayoría de las adolescentes y las jóvenes se encontraba en el nivel de estudios medio o secundario. Las jóvenes que referían un nivel de estudio superior se presentan en mayor porcentaje en las de nacionalidad chilena, con respecto de las extranjeras (36,02% y 18,54%, respectivamente, p=0,005). Las jóvenes en situación de convivencia con una pareja se presentaron en mayor porcentaje en la población migrante (21,49%), mientras que las jóvenes de nacionalidad chilena en su gran mayoría no reportaban ser casadas o estar en convivencia (88,23%, p=0,002). Las jóvenes adscritas a un sistema de salud previsional público o privado fueron en mayor porcentaje las chilenas (83,42% y 12,10%, respectivamente). Las jóvenes extranjeras reportaron en mayor porcentaje no disponer de ninguna previsión de salud (22,27%, p=0,001) (Tabla 2).

**Tabla 2:** Distribución de las variables sociodemográficas por nacionalidad en adolescentes y jóvenes en Chile

Variables	Categorías	Total (%)	Nacionalidad		p
			Extranjera (%)	Chilena (%)	
Nivel socioeconómico	Alto	6,88	5,84	6,92	0,204
	Medio	52,09	67,83	51,51	
	Bajo	41,03	26,33	41,57	
	Total	100	100	100	
Nivel de estudio	Superior	35,41	18,54	36,02	0,005
	Media	58,96	79,2	58,22	
	Básica	5,63	2,26	5,76	
	Total	100	100	100	
Situación de pareja	Conviviente	11,05	21,49	10,67	0,002
	Soltera	87,75	74,37	88,23	
	Casada	1,21	4,14	1,1	
	Total	100	100	100	
Salud previsional	Público	83,07	73,45	83,42	0,001
	Privado	11,83	4,28	12,1	
	Otros	1,4	0	1,46	
	Ninguno	3,69	22,27	3,02	
	Total	100	100	100	
Edad	Promedio	19,72	19,81	19,72	0,901
	(IC 95 %)	(19,58 - 19,86)	(18,36 - 21,26)	(19,58-19,86)	

Con relación a las variables reproductivas, las jóvenes extranjeras presentan en mayor porcentaje el antecedente de uno o más hijos/as (45,28%), mientras que en las jóvenes chilenas, la gran mayoría no refirió hijos/as (76,31%, p=0,002). Las jóvenes que usaron un MAC menos efectivo en la primera relación sexual fueron en mayor porcentaje las migrantes (69,83%), en tanto que las chilenas presentaron un mayor uso de MAC efectivo o muy efectivo (49,07%, p=0,034) (Tabla 5).

Las jóvenes extranjeras presentan mayor edad al momento de tener un embarazo no planificado con respecto a las adolescentes chilenas (18,92 vs. 17,67 años, p=0,033), con un promedio de 1,43 años más que las chilenas (Coef.: 1,43; IC 95%: 0,38-2,49) (Tabla 3).

**Tabla 3:** Distribución de las variables reproductivas por nacionalidad en adolescentes y jóvenes en Chile

Variables	Categorías	Total (%)	Nacionalidad		p
			Extranjera (%)	Chilena (%)	
Número de hijos	1 a 4	24,46	45,28	23,69	0,002
	Ninguno	75,54	54,72	76,31	
	Total	100	100	100	
Embarazo no planificado	No	81,83	76,04	82,05	0,326
	Sí	18,17	23,96	17,95	
	Total	100	100	100	
Uso MAC primera relación sexual	No	15,3	13,59	15,36	0,738
	Sí	84,7	86,41	84,64	
	Total	100	100	100	
Uso MAC última relación sexual	No	12,29	19,03	12,03	0,177
	Sí	87,71	80,97	87,97	
	Total	100	100	100	
Tipo de MAC usado en la 1° relación sexual	Efectivo, Muy efectivo	48,39	30,17	49,07	0,034
	Menos efectivo	51,61	69,83	50,93	
	Total	100	100	100	
Tipo de MAC usado en la última relación sexual	Efectivo, Muy efectivo	67,42	51,02	68	0,061
	Menos efectivo	32,58	48,98	32	
	Total	100	100	100	
Edad primer embarazo	Promedio	18,82	19,49	18,77	0,087
	(IC 95 %)	(18,59 - 19,05)	(18,70 - 20,28)	(18,53 - 19,01)	
Edad embarazo no planificado	Promedio	17,73	18,92	17,67	0,033
	(IC 95 %)	(17,40 - 18,05)	(17,82 - 20,03)	(17,34 - 18,0)	

En cuanto a las variables de sexualidad, la práctica de sexo anal se presenta en mayor porcentaje en las jóvenes de nacionalidad chilena (18,66%), mientras que las extranjeras en su gran mayoría no lo realiza (91,46%, p=0,040). Con referencia al tipo de pareja sexual en la primera relación, en las chilenas en su mayoría fue con una pareja andante, novio/a-polola/o, amigo/a (75,90%).

Por otro lado, la primera pareja sexual de las jóvenes extranjeras fue en mayor porcentaje su esposo o su conviviente (19,47%, p=0,017) (Tabla 4).

**Tabla 4:** Distribución de las variables de salud sexual por nacionalidad en adolescentes y jóvenes en Chile

Variables	Categorías	Total n(%)	Nacionalidad		p
			Extranjera n(%)	Chilena n(%)	
Inicio de actividad sexual	No	38,12	38,78	38,09	0,948
	Sí	61,88	61,22	61,91	
	Total	100	100	100	
Número de parejas sexuales últimos 12 meses	2 y más	22,31	23,16	22,28	0,908
	0 a 1	77,69	76,84	77,72	
	Total	100	100	100	
Uso de condón última relación sexual	No	46,51	57,32	46,08	0,195
	Sí	53,49	42,68	53,92	
	Total	100	100	100	
Antecedente de violencia de pareja	No	86,3	91,55	86,07	0,338
	Sí	13,7	8,45	13,93	
	Total	100	100	100	
Antecedente de aborto provocado	No	97,66	99,1	97,6	0,318
	Sí	2,34	0,9	2,4	
	Total	100	100	100	
Sexo oral	No	58,42	60,83	58,33	0,777
	Sí	41,58	39,17	41,67	
	Total	100	100	100	
Sexo anal	No	81,72	91,46	81,34	0,04
	Sí	18,28	8,54	18,66	
	Total	100	100	100	
Tipo de relación con primera pareja sexual	Recién conocido, familiar, abuso sexual	1,53	0,15	1,58	0,017
	Amigo o andante	16,27	12,35	16,42	
	Novio o pololo	75,61	68,03	75,9	
	Esposo, conviviente	6,58	19,47	6,09	
	Total	100	100	100	
Tipo de relación con última pareja sexual	Recién conocido, familiar, trabajadora sexual	0,75	0	0,78	0,076
	Amigo o andante	18,51	20,36	18,44	
	Novio o pololo	64,61	48,69	65,22	
	Esposo, conviviente	16,13	30,95	15,56	
	Total	100	100	100	
Edad de inicio actividad sexual	Promedio	16,31	16,74	16,29	0,093
	(IC 95 %)	(16,19 - 16,43)	(16,23 - 17,25)	(16,17 -16,41)	
¿Te has realizado alguna vez el test del VIH?	No	57,9	62,93	57,73	0,585
	Sí	42,1	37,07	42,27	
	Total	100	100	100	
¿Cuál es la principal razón por la que te hiciste el test del VIH?	Por mayor tranquilidad/exposición de riesgo	44,69	23,41	45,3	0,001
	Control o atención de salud	15,02	15,08	15,02	
	Control embarazo	38,1	45,23	37,89	
	Por motivos laborales	1,13	14,73	0,73	
	Dentro de una campaña de prevención	1,07	1,54	1,05	
	Total	100	100	100	

Las jóvenes chilenas en su mayoría se realizan el test del VIH por mayor tranquilidad o por exposición al riesgo (45,30%), mientras que en el caso de las jóvenes migrantes, las mayores frecuencias de toma del examen fueron por control de embarazo y por motivos laborales (45,23% y 14,73%, p=0,001) (Tabla 4).

La razón entre las jóvenes que no tuvieron sexo anal versus aquellas que sí lo tuvieron es 2,6 veces mayor en las jóvenes migrantes en comparación con las chilenas (OR: 0,38; IC 95%: 0,16-0,91).

La razón entre las jóvenes que usaron un tipo de MAC menos efectivo en la primera relación sexual versus aquellas que usaron un MAC efectivo o más efectivo, es 2,19 veces mayor en las jóvenes migrantes en comparación con las jóvenes chilenas (OR: 2,19; IC 95%:1,03-4,64).

La razón entre las jóvenes que usaron un tipo de MAC menos efectivo en la última relación sexual versus aquellas que usaron un MAC efectivo o más efectivo, es 2,40 veces mayor en las jóvenes migrantes en comparación con las jóvenes chilenas (OR: 2,40; IC 95%:1,16-4,97) (Tabla 5).

**Tabla 5:** Odds ratio (OR), coeficiente (Coef.) entre la variable nacionalidad y variables de salud sexual y reproductiva en adolescentes y jóvenes en Chile

Migrante	Salud sexual y reproductiva			
	Edad embarazo no planificado	Sexo anal	Tipo de MAC usado en la primera relación sexual	Tipo de MAC usado en la última relación sexual
	Coef.a	ORa	ORa	ORa
	IC 95 %	IC 95 %	IC 95 %	IC 95 %
Si	1,43	0,38	2,19	2,4
	(0,38 - 2,49)^**^	(0,16 - 0,91)^*^	(1,03 - 4,64)^*^	(1,16 - 4,97)^*^
No	1	1	1	1

Regresión lineal múltiple (Coefa.: Coeficiente, ajustado por edad, nivel socioeconómico y nivel de estudio), regresión logística múltiple, ORa, odds ratio, ajustado por edad, nivel socioeconómico y nivel de estudio; IC: intervalo de confianza. Nacionalidad (0 = Chilena, 1= Migrante). Edad embarazo no planificado (edad en años cumplidos). Sexo anal (0 = No, 1 = Sí), Tipo de MAC usado en la primera relación sexual (0 = Efectivo, Muy efectivo, 1 = Menos efectivo), Tipo de MAC usado en la última relación sexual (0 = Efectivo, Muy efectivo, 1 = Menos efectivo). ^*^p < 0,05, ^**^p < 0,01.

## DISCUSIÓN

En la última década se ha observado un aumento sostenido de las migraciones a nivel regional e internacional, lo que ha presionado a los gobiernos para generar estrategias de inclusión de poblaciones extranjeras 2,4. El sistema sanitario se ha visto particularmente presionado para generar medidas que aseguren el acceso a la salud de poblaciones migrantes 1. Sin embargo, hay grupos específicos, como el de las jóvenes y las adolescentes, que debido a su volumen inferior en relación con grupos de adultos tiende a ser relegado 7,11. Estas jóvenes y adolescentes llegaron en la etapa de adolescencia. Esto es relevante si se considera que la adolescencia en sí misma es una etapa de mayor vulnerabilidad por los múltiples cambios biopsicosociales que se experimentan en un periodo corto de tiempo, sumado al estrés que enfrentan las adolescentes y las jóvenes migrantes al dejar su país de origen e insertarse en otro. Esto supone que muchas de estas jóvenes se encuentran vivenciando el duelo migratorio y una adecuación cultural, lo que las hace más sensibles a problemáticas de salud 7,10.

Las jóvenes migrantes que viven en Chile representan un porcentaje menor del total de las adolescentes y jóvenes del país, y las nacionalidades más prevalentes son la venezolana y la peruana, tendencias que coinciden con las estadísticas migratorias del país 4.

En cuanto a las diferencias sociodemográficas, se observó un mayor porcentaje de jóvenes chilenas en nivel educacional superior que las extranjeras, lo cual contrasta con los datos de la Encuesta de Caracterización Socioeconómica Nacional (CASEN), los cuales muestran que las mujeres extranjeras mayores de 18 años tienen en promedio dos años más de escolaridad que las mujeres nacidas en Chile 27. Por otro lado, fue más frecuente encontrar jóvenes casadas o en convivencia entre las extranjeras. Esto sugiere que muchas mujeres jóvenes extranjeras, una vez terminados sus estudios secundarios, rápidamente se insertan en el ámbito laboral o familiar, donde el primero toma especial relevancia, ya que es una de las principales razones para migrar, por mejores condiciones de vida 1. Estos datos se relacionan con estudios sobre la identificación de adolescentes y jóvenes migrantes, que señalan que estos deben iniciar una vida de adultos más tempranamente que sus pares no migrantes 7,8.

Un aspecto preocupante desde la protección de la salud, es que una de cada cinco jóvenes extranjeras no estaba afiliada al sistema previsional, mayor a lo informado por la encuesta CASEN para población nacida fuera de Chile 27. Esto las ubica en un estado de desprotección para el cuidado de su salud, incluida la SSR, a pesar que el Estado de Chile ha impulsado diferentes estrategias que aseguren el acceso a la salud de personas migrantes, especialmente menores de edad 6. La mayoría de las mujeres migrantes que logran entrar en contacto con el sistema de salud, lo hacen a través del control gestacional, el parto y el puerperio 5. Así, en las jóvenes provenientes de otros países, la principal razón para realizarse el test del VIH fue el control de embarazo, lo que se correlaciona con el mayor número de hijos que refieren, mientras que para las chilenas fue para mayor tranquilidad o por exposición a riesgo. Llama la atención que la toma del examen del VIH sea considerablemente mayor por motivos laborales en el caso de las jóvenes migrantes, lo que podría indicar que estas mujeres se encuentran mayormente insertas en el ámbito laboral, o que se les solicita el examen más que a las chilenas al momento de ingresar a algún empleo. Esto último sería un hallazgo que transgrediría la normativa legal vigente (Ley 19779, normas relativas al virus de inmunodeficiencia humana), la cual establece que no se puede exigir la realización de este examen ni condicionar la contratación de las trabajadoras al resultado de éste. Por otro lado, no se encontraron diferencias en la frecuencia de la toma del examen y los conocimientos sobre las vías de transmisión del VIH. En ambos grupos, menos del 30 % presentó un conocimiento totalmente correcto, tampoco se encontraron diferencias para el uso de condón en la última relación sexual. Estas similitudes podrían tener relación con el nivel socioeconómico comparable entre los grupos, el cual está asociado de manera importante con los grados de conocimientos del VIH 28. Estos resultados indican la necesidad de seguir fortaleciendo las estrategias de prevención del VIH focalizadas en población adolescente y joven.

Por otro lado, se hace necesario conocer, desde las perspectivas de las mismas jóvenes, sobre las barreras de acceso a otros servicios de SSR, para generar estrategias específicas con enfoque de género y pertinencia cultural, más allá de lo relativo al cuidado materno-infantil 5,22.

Para las variables reproductivas, las jóvenes migrantes refirieron más frecuentemente el antecedente de tener hijos/as que las chilenas, lo cual es coherente con las tasas globales de fecundidad de la región, en donde países como Haití y Bolivia están sobre 2,5 y en Chile es 1,6, similar a la de Brasil 20. Sin embargo, no presentaron más embarazos en el periodo de la adolescencia, lo cual difiere con otros estudios que muestran que los embarazos antes de los 20 años son más frecuentes en las mujeres migrantes 29. Estas diferencias podrían deberse a diversos factores socioculturales, como por ejemplo a la mayor necesidad de generar redes familiares sólidas. En consecuencia, se hace necesario comprender las motivaciones, las representaciones y las prácticas culturales asociadas a la maternidad en las mujeres migrantes 30.

En cuanto la frecuencia de uso de anticonceptivos, no se encontraron diferencias, lo cual muestra que las jóvenes migrantes no están en desventaja con respecto de las chilenas para acceder a anticonceptivos, esto coincide con un estudio realizado en Colombia en mujeres migrantes venezolanas 30. En cuanto al MAC, se observó una tendencia al uso de métodos más efectivos entre la población chilena con respecto a la migrante, lo que puede estar relacionado con las creencias en torno a la fertilidad, la anticoncepción y las barreras e inequidades de acceso a anticonceptivos modernos, ya sea en su país de origen o a su llegada a Chile 11.

Los comportamientos en sexualidad como la edad de inicio sexual, el número de parejas sexuales y el uso de condón fueron similares en ambos grupos. Las prácticas de sexo oral y anal son cada vez más comunes entre jóvenes y adolescentes; no se observaron diferencias para la práctica de sexo oral en las jóvenes chilenas en comparación con las migrantes, mientras que el sexo anal es más practicado por las chilenas, similar a lo reportado en otros estudios 21. En cuanto al tipo de pareja sexual, en la población extranjera fue más frecuente que se tratara del esposo o conviviente 21. Las diferencias entre las variables sexuales de las jóvenes chilenas y las extranjeras pueden tener relación con lo observado en estudios sobre el comportamiento sexual de riesgo de adolescentes y jóvenes latinas en Estados Unidos. Estos muestran que las primeras generaciones de migrantes tienen comportamientos menos riesgosos que las generaciones posteriores, probablemente por la fuerte influencia cultural conservadora de su país de origen y la aculturación posterior 10,17,31. Esto es algo que se deberá investigar, especialmente por la transformación social que está viviendo Chile en temas de sexualidad, como el movimiento feminista y las discusiones sociales y legislativas en temas como el aborto libre, el matrimonio igualitario, las leyes de identidad e igualdad de género, entre otras, que impactarán en las decisiones sexuales y reproductivas de las personas, especialmente de adolescentes y jóvenes.

El presente estudio buscó ser un aporte frente a la escasa evidencia con respecto a la SSR de jóvenes y adolescentes extranjeras de la región en Chile, sin embargo, hay limitaciones relacionadas con el dinamismo en torno a los datos migratorios, y la dificultad de llevar registros, en la medida en que el ingreso por paso no habilitado y la dificultad para regularizar la situación migratoria van en aumento, fenómeno observado en diferentes partes de la región. A esto se suman las limitaciones debido a la utilización de una fuente secundaria de muestras ponderadas; no están representadas poblaciones específicas, como la migrante, lo que disminuye la potencia estadística al impedir la utilización de ciertos estadígrafos, además de la imposibilidad de profundizar en las diferencias de este heterogéneo grupo. Cabe señalar que la variable nacionalidad, utilizada para identificar a jóvenes migrantes, no permite identificar a aquellas jóvenes chilenas hijas de extranjeros, quienes probablemente habitan en condiciones de vulnerabilidad con mayor pobreza multidimensional que la población chilena 27, vivenciado la limitación de oportunidades y el estigma de la población migrante.

Las jóvenes extranjeras son un grupo pequeño dentro del grupo de adolescentes y jóvenes que viven en Chile. Existen algunas similitudes entre las adolescentes y jóvenes migrantes y chilenas en cuanto al acceso a anticonceptivos y diferencias en torno al tipo de pareja y estado civil al momento del inicio de las relaciones sexuales, el número de hijos, la práctica de sexo anal y el tipo de anticonceptivo usado. Dado el aumento sostenido de las migraciones en la región de las Américas y en el mundo debido a catástrofes naturales y sociopolíticas, es necesario generar políticas específicas e integrales para el grupo de jóvenes y adolescentes migrantes, que tienden a ser invisibilizadas frente a políticas focalizadas en otros grupos considerados prioritarios: niñeces y gestantes. El abordaje integral no es responsabilidad exclusiva de los sistemas sanitarios, sin embargo, es necesario la realización de estudios, tanto cualitativos como cuantitativos, para identificar las brechas en salud que viven las jóvenes extranjeras y tomar medidas que tiendan a su inclusión al sistema sanitario ♠
